# Validation of a clinical competence evaluation tool for community service nurses in North West province, South Africa

**DOI:** 10.4102/hsag.v26i0.1602

**Published:** 2021-11-12

**Authors:** Kholofelo L. Matlhaba, Abel J. Pienaar, Leepile A. Sehularo

**Affiliations:** 1 School of Nursing Science, Faculty of Health Sciences, North-West University, Mahikeng, South Africa; 2 Department of Health Studies, College of Human Sciences, University of South Africa, Pretoria, South Africa; 3 Department of Psychology, Faculty of Health Studies, University of Venda, Thohoyandou, South Africa; 4 Department of Graduate and Research, Shifa College of Nursing, Shifa Tameer-e-Millat University, Islamabad, Pakistan; 5 Faculty of Health Sciences, North-West University, Mahikeng, South Africa

**Keywords:** clinical competence, evaluation tool, experts, reliability, validation

## Abstract

**Background:**

Little has been done to evaluate clinical competence of community service nurses (CSNs) during the 12-month compulsory community service in South Africa. Evaluating clinical competence of CSNs would be of benefit as it might improve quality patient care and promote patient satisfaction. It therefore became of paramount importance for the researcher to establish some method of evaluating the CSNs’ clinical competence during their compulsory service in the North West province (NWP), South Africa.

**Aim:**

To evaluate the clinical competence evaluation tool (CCET) for CSNs for reliability and validity.

**Setting:**

A selected regional level 2 hospital.

**Methods:**

Ten experts participated in the validation process. The tool was tested at one of the public hospitals in the NWP and 11 out of 13 CSNs participated in this process. Statistical Package for the Social Sciences version 25 was employed and the reliability of the tool was measured using Cronbach’s alpha.

**Results:**

This tool’s content validity index has exceeded 0.80 and is indicated at 0.98, which reflects excellent content validity. The higher the content validity ratio score the greater the agreement amongst the experts. The Cronbach’s alpha coefficients in the six competencies are all greater than 0.7 implying that the tool developed in this study is reliable. All the experts indicated that the tool is clear, simple, general, accessible and important.

**Conclusion:**

From the above-mentioned results, a CCET for CSNs was proven to be valid and reliable.

**Contribution:**

This was the first tool to be developed in NWP of South Africa.

## Introduction

The South African Nursing Council (SANC) stipulates under the *Nursing Act* (33 of 2005) that the mission of SANC (2005) is to:

[*S*]afeguard the health and well-being of the public by ensuring that nurses and midwives keep their skills and knowledge up to date and uphold the standards of their professional code. (p. 2)

Further SANC (2005) states that:

[*N*]urses who acquire the knowledge, skills and behaviours that meet our standards will be equipped to meet changing needs, developments, priorities and expectations in health and healthcare, improve health and well-being and drive up standards and quality, working in a range of roles including practitioner, educator, leader and researcher. (p. 2)

Therefore, newly qualified nurses are assisted with orientation and transition support programmes to make the transition through the working environment (Hussein et al. 2017). During this transition phase, they are expected to become competent practitioners ready to meet the real-life challenges of the healthcare system.

In South Africa, since 2008 it is mandatory for nurses who have qualified as a Nurse (General, Psychiatric and Community) and Midwife leading to registration in Government Gazette Notice No. R425 of 22 February 1985 to complete 12-months’ community service before they can be registered as professional nurses. During this community service period, community service nurses (CSNs) are allocated to different public healthcare facilities. However, since the inception of this community service, little has been done to evaluate their clinical competence, despite it being expected of them to be competent practitioners when resuming their duties as professional nurses. It was therefore of paramount importance for the researcher to establish an instrument to evaluate their clinical competence during their compulsory service, hence the need to evaluate the clinical competence evaluation tool (CCET) for CSNs for reliability and validity.

Competence is regarded as the:

[*A*]bility to perform a work-role to a defined standard with reference to real working environments that ideally includes a person’s ability to demonstrate their cognitive knowledge, skills, behaviours and attitudes in any given situation. (Franklin & Melville 2015:26)

Wu et al. (2015) described clinical competence as the ‘theoretical and clinical knowledge used in the practice of nursing, incorporating the psychomotor skills and problem-solving ability with the goal of safely providing care for healthcare users’. Benner (1984) emphasised that as nurses’ progress through various levels of ability their clinical competence develops over time. It is noteworthy to mention that this research focuses predominantly on clinical competence of CSNs. Therefore, for the purpose of this research, clinical competence is considered the abilities of the CSNs to ‘work competently in providing quality nursing care to the patient under their care during community service period’ (Matlhaba, Pienaar & Sehularo 2019). For the 12-months community service period, CSNs are allocated in healthcare facilities such as clinics and hospitals and expected to perform delegated duties under the supervision of experienced professional nurses (Matlhaba et al. 2019).

From the literature, it can be established that several CCETs for newly qualified nurses already exist including those of Nilsson et al. (2014), Liou and Cheng (2014), Safadi et al. (2010), Cowan et al. (2008) and Liu et al. (2007). However, as established from the literature, there are no CCETs specifically developed for CSNs in South Africa (SA). The lack of an evaluation tool for clinical competence of CSNs prompted the researcher to develop a CCET in the North West province (NWP). The tool consists of five sections. Section A consists of six main competencies, 17 domains and 144 items to be completed by the CSN using a 5-point Likert scale. The main competencies are legal practice, ethics and professional practice, operational (unit) management and leadership, contextual clinical and technical competence, therapeutic environment and quality nursing care. Sections B–D consists of semi-structured questions. Section B to be completed by the community service himself or herself (self-evaluation), Section C to be completed by a fellow CSN (peer-evaluation) and Section D to be completed by a registered or professional nurse (mentor-evaluation). Section E to be completed by the CSN (self-learning assessment).

## Research purpose

The purpose of the research is to evaluate the CCET for CSNs for reliability and validity.

### Research design

This research utilised a mixed method with multiphase. According to Creswell (2014:266), the justification for a mixed method research is that both qualitative and quantitative research collectively delivers an improved and stronger understanding of a research problem or issue than if either research approach was applied individually.

#### Population and sample

Gray, Grove and Sutherland (2016) defined a population as ‘all the components, including characters, objects or elements that meet certain criteria for inclusion in a particular world’. Similarly, Grove, Gray and Burns (2015) and Polit and Beck (2017) agreed that population is a ‘collection of objects, events or individuals’ with the same mutual ‘characteristics that the researcher is interested in studying’ or the ‘aggregate of all cases that conform to some designed set of specifications’. A sample then is a portion of the target population selected to participate in the research study (Grove et al. 2015). The population for this section of the study was expert in nursing education and nursing practice and the CSNs in NWP who were engaged with their community service during data collection between July and October 2019.

#### Sampling of experts

The target population for validation of the tool was 15 experts. However, five potential participants declined to participate and the researcher respected their decision for ethical reasons. Ten experts participated in the validation of the tool. Inclusion criteria for validation of the tool were:

- Different nursing experts from various provinces of South Africa. Experts who participated in this study were from North West, Gauteng, Limpopo and KwaZulu-Natal provinces.
- Experts registered as such with SANC.
- Experts who were directly and indirectly involved in nursing education, training and practice.
- Experts who were nurse educators, leaders or managers.

This study excluded nurses who were not registered and recognised as experts by SANC such as student, assistant and staff nurses. The study also included non-nursing professionals.

#### Sampling of community service nurses

The researcher used a purposive-sampling technique to select CSNs for participation in the study. The researcher received a list of all CSNs from the selected regional hospital’s training coordinator, who were 13 in total. These CSNs were placed at the hospital for more than 6 months of their community service. All 13 participants who were placed at a selected regional hospital were approached by the researcher for participating in the study and only two did not return their responses and the researcher respected their decision. This means that 11 CSNs from one regional level 2 hospital participated in the study. This means 84.6% of the participants participated, which shows a good response rate. The purpose of the study and what was expected was explained and participants had the right to agree to complete the tool or decline. Participants were given the tool to complete and return after a week. Initially, 13 tools were provided where CSNs were requested to evaluate their clinical competence by completing Section A (self-evaluation) of the CCET using the 5-point Likert scale, however, 11 of the 13 tools were received back. The returned and completed tools were analysed.

### Data collection

A drafted CCET was sent to the 10 experts who agreed to participate in the study through the email. All experts were given a minimum of a week to return the evaluated tool back to the researcher and all experts returned it within five working days. Community service nurses were also given the tool to complete and return after a week. Initially, 13 tools were provided where CSNs were requested to evaluate their clinical competence by completing Section A (self-evaluation) of the CCET using the 5-point Likert scale, however, 11) of the 13 tools were received back. Ten tools received from the experts and11 CSNs were analysed by the researcher and the statistician.

### Ethical considerations

Approval for this study was first obtained from the scientific committee of the School of Nursing Science (SONS), Faculty of Agriculture, Science and Technology (FAST) ‘Health Science Ethics Committee’ (HSEC) of the North-West University (Reference Number: NWU-00230-18-A9). For the study’s duration, the following fundamental ethical principles as stipulated by Smit (2018) were ensured:

**Beneficence:** The well-being of the participants was secured at all times. Participants were informed of their right to participate and to withdraw anytime they wish to without any fear of punishment. Participants were protected from any harm or discomfort. Even though this study did not involve harmful intervention to participants, there was no manipulation of participants.

**Justice:** During this study, participants’ right to fair selection and treatment was ensured. Participants’ cultural values and time agreed upon between the researcher and participants were respected. There were no incentives for participants and they were made aware before the data collection process commenced.

Rights to privacy, confidentiality and anonymity was ensured. Confidentiality was maintained, as the collected data were only made available to the research team. Care was taken to ensure that the privacy and anonymity of the participants and their identities were protected; the researcher used identity codes to identify the participants during data collection.

**Rights to informed consent:** Participants received detailed consent with sufficient information about the research and were informed about their rights to participate voluntarily or to decline any participation. A written informed consent form was signed prior to participation. Participants who declined to give written consent were excluded from the study participation.

### Data analysis and discussions

To measure the tool’s reliability and validity, a content validity index (CVI), content validity ratio (CVR) using the experts’ validation and the Cronbach’s alpha was performed using the Statistical Package for the Social Sciences (SSPS) version 25. After obtaining a written approval from potential participants, 10 experts and 11 CSNs participated in this process, respectively. Validity is the degree to which an instrument measures what it is supposed to be measuring (Polit & Beck 2017). Content validity refers to the sampling adequacy of items for the construct that is being measured (Polit & Beck 2017). Reliability is the degree of consistency or precision with which an instrument measures an attribute (Polit & Beck 2017). According to Polit and Beck (2017) the higher the reliability of an instrument the lower the error in obtained scores.

This tool’s CVI has exceeded 0.80 as it is at 0.98, which shows excellent content validity as it is suggested that CVI exceeding 0.80 is preferred (Polit & Beck 2017). Formula put of CVI and CVR were used to achieve this score. The Cronbach’s alpha coefficients in the six competencies are all greater than 0.7 and this implies that the tool used for this study was proven to be reliable during the study. The higher CVR score indicates greater agreement amongst panel members (Gilbert & Prion 2016). All experts and CSNs indicated that the tool is clear, simple, general, accessible and important.

### Analysis from the validation process

The CVI and CVR processes and the Cronbach’s alpha using the SPSS version 25 programme were used for both the experts and CSNs. Descriptive analysis of CVI and CVR (Quality and Validity and Reliability) scores were calculated. The tool was found to be understandable, easy to complete and most likely to be used by all experts. Experts also found that the tool was appropriate and will contribute to the professional development of the CSNs in preparation of their roles as professional nurses after completion of the community service. Except for the statistical evidence, some experts also qualified their score by adding remarks at the end as stated here:

‘I found the tool being sufficient enough to guide, assess and assist in building and grooming the future nurses to become those that the profession can be proud of. Essentials are vital and I have found them to be enough and non-complicated.’ (Expert 5, female, age above 45, PhD, Government)

‘The tool is very relevant and displays the competencies needed after completion of the community service.’ (Expert 1, female, age 31–35, PhD, University)

Adding to the following some experts suggested that the evaluation intervals should be performed on a quarterly basis. This is what they indicated:

‘The evaluation of community service nurse practitioner within a week of commencement of community service appears too soon. One would recommend that the evaluation be done on a quarterly basis (every three months).’ (Expert 7, age above 45, PhD, Government)

‘CSN to be evaluated for competency on quarterly basis, preferably after every 3 months because one can only be partially competent with practice, CSN have little exposure, if not none, to management of the unit or to the ordering of ward equipment such as drugs, dry/wet dispensary, stationery, et cetera.’ (Expert 10, female, age above 45, Masters, College)

Even though all of the experts rated the tool as essential and useful, some experts expressed their concern regarding its length. Here is the evidence from experts’ feedback:

‘The tool is quite long and has the potential of not being fully completed. The layout of the tool might be the reason why it looks quite long.’ (Expert 2, female, age above 45, Masters, University)

‘The tool is comprehensive but once used you might find areas will need amendment. In some instances, nurses might object to the length of it.’ (Expert 3, female, age above 45, PhD, Governance)

However, whilst not dismissing the given comments, Persky and Robinson (2017:72) stated that experts develop through the years of experience and by progressing from novice, advance beginner, proficient, competent and finally expert. These stages are contingent on progressive problem solving, which means individuals must engage in increasingly complex problems, strategically aligned with the learner’s stage of development. Based on the above-mentioned statement, it can therefore be deduced that CSNs cannot be expected to be completely competent immediately after commencement of the community service, as they have no experience in many of the situations in which they are expected to perform. Community service nurses might lack the competence to demonstrate safe nursing practice and require continual verbal and physical cues (Benner 1984). Therefore, several meetings with the mentor according to the CCET could improve the level of competence of the CSNs, who will be preparing themselves for a professional nurse’s role after completion of the 12-months period.

The other concern raised by three experts is that the tool is evaluating clinical competence of CSNs in all disciplines, whereas some CSNs are not placed in those disciplines during their community service. This statement was supported by the results of objective 1 and 2 of this study, where some CSNs and PNs mentioned the challenges or discrepancies with CSNs’ allocations in the facilities (Matlhaba et al. 2019). However, the researcher envisions that in the near future, specific CCETs can be developed where the focus will be on specific disciplines such as mental-health nursing and maternal and neonatal care. Responding to the comments, it is common cause that the CSN qualifies in all disciplines (R425); therefore the baseline assessment should be carried out in all disciplines.

Tables 1–4 depict the demographics of experts, results of overall content validity of the CCET and results of content validity of the CCET for each domain, respectively.

**TABLE 1:** Demographic characteristics of experts.

Demographic characteristics of experts	Variable	Value
Number of experts	-	10
Age group (years)	< 30	1
	35‒65	9
Gender	Female	7
	Male	3
Qualification	PhD	5
	Masters	4
	Degree	1
Work experience (years)	< 10	1
	10–20	1
	21–30	3
	> 30	5
Area of specialty	Academic (University)	2
	Academic (College)	1
	Governance (SANC)	1
	Government (NWDoH)	1
	Government (LDoH)	1
	Government (Hospital)	12
	Professional Association	1
	Labour movement	1

PhD, Doctor of Philosophy; SANC, South African Nursing Council; NWDoH, North West Department of Health; LDoH, Limpopo Department of Health.

**TABLE 2:** Results of content validity of the clinical competence evaluation tool for each domain.

Domain	Essential or useful domains (*n*)	Not necessary domains (*n*)	Unrated domains	Total number of experts	Essential or useful domains (CVR)
1	10	0	0	10	1
2	10	0	0	10	1
3	10	0	0	10	1
4	10	0	0	10	1
5	10	0	0	10	1
6	10	0	0	10	1
7	10	0	0	10	1
8	10	0	0	10	1
9	10	0	0	10	1
10	9	1	0	10	0.8
11	10	0	0	10	1
12	10	0	0	10	1
13	10	0	0	10	1
14	10	0	0	10	1
15	10	0	0	10	1
16	10	0	0	10	1
17	10	0	0	10	1

Note: CVI = (Ne – N/2)/(N/2), where Ne is the number of experts identifying an item as ‘essential’ and N is the total number of experts (N/2 is half the total number of experts).

CVR, content validity ratio.

Content validity index (CVI): 0.98.

**TABLE 3:** Results of overall content validity of the clinical competence evaluation tool.

CCET	Rated tool as essential or useful	Rated tool as not necessary	Unrated tool	Total number of experts	Essential or useful domains (CVR%)
CCET	10	0	0	10	1

Note: CVR = (Ne – N/2)/(N/2) where Ne is the number of experts identifying an item as ‘essential’ and N is the total number of experts (N/2 is half the total number of experts).

CCET, clinical competence evaluation tool; CVR, content validity ratio.

Content validity ratio of overall tool: 1.

**TABLE 4:** Reliability analysis.

Subscale (competencies)	Cronbach’s alpha (α)	No. of Items	Internal consistency
Legal practice	0.919	15	Excellent
Ethics and professional practice	0.900	14	Excellent
Operational (unit) management and leadership	0.901	19	Excellent
Contextual clinical and technical competence	0.963	81	Excellent
Therapeutic environment	0.892	9	Good
Quality nursing care	0.808	6	Good

**Total**	**0.971**	**144**	**Excellent**

From Table 1, this study complied with the suggestions by Gilbert and Prion (2016) on sampling of experts. According to Gilbert and Prion (2016), the content evaluation panel should be composed of persons who are experts in the domain being studied. Ideally, there should be a range of experts (also known as subject matter experts) on this panel at various professional levels. In content areas where it is difficult to find experts, the use of three experts is acceptable; normally, a panel of 5–10 experts is preferred (Gilbert & Prion 2016).

In this study, Table 2 personifies the results of content validity of the CCET for each domain.

Table 2 presents the results from the CVI. As mentioned previously, each of the experts was supplied with the CCET (Table 2) and the instruction form. Each expert was asked to rate each of the items as ‘essential’, ‘useful’ or ‘not necessary’.

Table 3 presents the results from the CVR. As part of the instructions to the experts, each of the experts was supplied with the CCET and the instruction form. Each expert was asked to rate each of the items in the tool as ‘very likely to be utilised’, ‘somewhat likely to be utilised’, ‘likely to be utilised’ and ‘unlikely to be utilised’.

Gilbert and Prion (2016) suggested that when all the experts say that the tested skill is ‘essential’, or when none of the experts says the skill is ‘essential’, the researcher can be confident to include or delete the particular item. It is when there is no consensus amongst the experts that item issues arise. The authors further suggest that two assumptions are made, each of which are consistent with established psychophysical principles:

- Any item, performance that is perceived to be ‘essential’ by more than half of the experts has some degree of content validity.
- The more experts (beyond 50%), perceiving an item as ‘essential’, the greater the extent of its content validity (Gilbert & Prion 2016).

Lawshe (1975) cited in Ayre and Scally (2014) suggested that CVR values range between –1 (perfect disagreement) and +1 (perfect agreement) with CVR values above zero indicating that over half of panel members agree that an item is essential. Therefore, in this study, all 10 of the experts rated the tool as essential and useful, likely and very likely to be used, hence the CVR value for this tool is 1, which indicates perfect agreement between the experts. No items were deleted from the tool.

### Analysis of results from reliability testing process

Pilot testing was carried out to test the reliability of the tool. As a result of time constraints, the results were established from a sample size of 11 participants, which the researchers observed to be a limitation but one that provides opportunities for further research.

### Section A: Demographic information

The sample size consisted out of a total of 11 participants (*N* = 11).

#### Gender profile

The majority of the participants were females (8 = 72.7%) and a third of the participants are males (3 = 27.3%). The demarcation is an indication that more females feature in the nursing profession. This reflection concurs with the results of the study conducted by Ogunyewo et al. (2015) in Nigeria. The study reported that 83.33% of participants who chose nursing as a career were female; furthermore, 91.25% of participants agreed that nursing is primarily for women (Ogunyewo et al. 2015). Supporting the above-mentioned notion, Zamanzadeh et al. (2013) stated that men because of male gender characteristics and existing public image do not often consider nursing as a career choice. According to Zamanzadeh et al. (2013), the feminine nature of nursing has been so prevalent that the caring image of the profession has been used to symbolise the epitome of femininity. Therefore, nursing is regarded as a feminine profession around the globe. In addition, the SANC in the *Nursing Act* 33 of 2005) asserted that there are more female than male nurses in their annual statistical records.

#### Age group profile

Majority of the participants were 30 years old and below, which could be a clear indication that those admitted to the 4-year nursing training programmes are amongst the youth. It could also be a reflection that nursing is perceived to be attractive to school leavers. This notion is supported by existing literature on the perceptions of nursing as a career of choice. The results of the studies conducted by Mohamed and El-Sayed (2013) in Egypt and Rajasree (2016) in Saudi Arabia reveal that 89.39% and 85.5% of participants, respectively, had positive perceptions of nursing as a career. In the study conducted by Ogunyewo et al. (2015) in Nigeria, 46.25% of participants were willing to consider nursing in the future.

#### Highest educational qualification obtained profile

With regard to highest educational qualification, only a third of the participants are diploma holders. The difference in the participants’ percentage in this study can be attributed to the fact that there is an increase in public recognition of degree-qualified nurses rather than diploma-qualified nurses. This is seen in many countries including Canada, Australia, New Zealand, Norway, Spain and others where a bachelor’s degree is a requirement for entry into professional nursing (Aiken et al. 2014). However, in SA there is currently no differentiation between degree-qualified and diploma-qualified nurses in the clinical practice (Roets, Botma & Grobler 2016). The duration of both the degree and diploma programmes is 4 years. On completion of the 4-year training, both the degree and diploma graduate nurse will be registered with SANC as a general, community health and psychiatric nurse and midwife after completion of their 12-months community service.

#### Province of training profile

From the results, one out of the 11 participants completed nursing training in Limpopo province. The allocation of this participant in the NWP was because of the fact that CSNs can be assigned anywhere across the country and not automatically in their province of training.

### Section B: Reliability analysis

Cronbach’s alpha (α) reliability coefficient whose numerical value ranges from zero to one, measures the reliability (or internal consistency) of a questionnaire consisting of Likert-type scales and items. A high value (close to 1) for Cronbach’s alpha reliability coefficient indicates good internal consistency of the items in the scale.

The Cronbach’s alpha coefficients in Table 4 are all greater than 0.7 and this implies that the questionnaire used for this study was proven to be reliable. It therefore indicates that the variables cited here are appropriate for exploratory factor analysis (EFA). Exploratory factor analysis refers to a factor analysis undertaken to explore the underlying dimensionality of a set of variables (Polit & Beck 2017). It further confirms that similar results would always be produced for repeated study elsewhere. Grove et al. (2015) confirmed that if variables are reliable, results will be consistent.

### Section C: Descriptive statistics (competencies)

**FIGURE 1:**
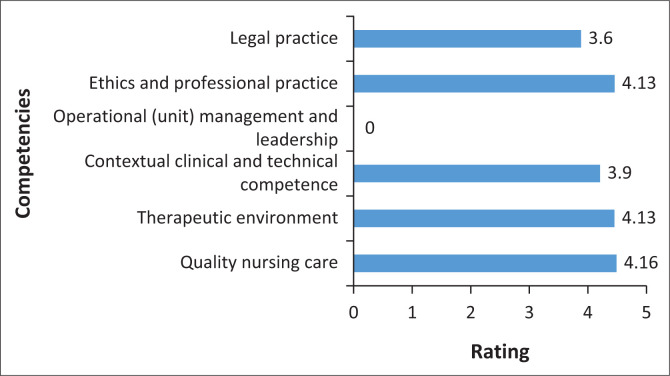
Graphical display of competencies overall ratings (Means).

Descriptive statistics refers to the statistics used to describe and summarise data (e.g. mean and percentage) (Polit & Beck 2017). Figure 1 represents the graphical display of competencies overall ratings. The following rating scale was used: Scale: 1 = novice, 2 = advanced beginner, 3 = competent, 4 = proficient, 5 = expert.

#### Legal practice

The overall mean for legal practice was established to be 3.60. The given information reflects that the participants were competent in matters that relate to their legal mandates with reference adherence of legal standards applicable to the nursing profession.

#### Ethics and professional practice

The overall mean rating for ethics and professional practice is 4.13 and is a reflection that the participants where proficient in their adherence to ethical and professional conduct of the nursing profession.

#### Operational (unit) management and leadership

The overall mean rating for operational (unit) management and leadership was 3.51. Even though these results reflect that participants were competent, the researcher suggests that the score could have been higher if they were given opportunities to take charge and run the ward during community service.

#### Contextual clinical and technical competence

The overall mean rating for contextual clinical and technical competence rated was 3.90. It should be observed that this section of the CCET comprises three sub-sections, namely Medical/Surgical Nursing, Midwifery and Mental Health Nursing. The level of competence for CSNs is dependent on the length of time they spend in a particular unit or ward. In this instance, medical or surgical nursing items rated high scores followed by postpartum care.

#### Therapeutic environment

The overall mean rating for therapeutic environment was 4.13, which is a reflection that the participants where proficient in creating a healthy and safe environment for self, healthcare users, families and colleagues.

#### Quality nursing care

The overall mean rating for quality nursing care was 4.16, which reflects a high level of competence. This result proves that participants have the ability to provide comprehensive or excellent nursing care for the healthcare user under their care.

### Section D: Correlation analysis

#### Spearman’s rank rho test

This test is concerned with the correlation between two ranked variables (X and Y). The correlation is statistically significant if the *p*-value is less than 0.05 level of significance.

The coefficient of Spearman’s rank correlation is given by Equation 1:


$$r=1-\frac{6\sum d^{2}}{n\left(n^{2}-1\right)}$$ [Eqn 1]

where:

*d* = differences of ranks of corresponding values of X and Y.

*n* = number of paired values in the data –1 ≤ *r* ≤ 1.

Table 5 summarises the correlations between the six competencies of the CCET. Correlations are associations or bonds between variables and the correlation coefficient refers to an index summarising the degree of relationship between variables (Polit & Beck 2017). When there is an association between two variables, the average value of one variable changes as the value of the other variable is changed (Bracken 2014). A correlation coefficient ranging from +1.00 indicates a perfect positive relationship, through 0.0 there is no relationship and to –1.00 there is a perfect negative relationship (Polit & Beck 2017). As all the *p*-values (probability values) in Table 5 are greater than 0.05 (5%) level of significance, then the CCET competencies are not significantly correlated. Each variable is independent in its own nature and does not influence the impact of another.

**TABLE 5:** Correlations amongst the clinical competencies (*N* = 11).

Competencies	Legal	Ethics	Operational	Contextual	Therapeutic	Quality
**Legal**						
Correlation coefficient	1	0.492	0.478	0.536	0.575	0.221
*p*	-	0.124	0.137	0.089	0.064	0.514
**Ethics**						
Correlation coefficient	0.492	1	−0.199	0.419	0.431	−0.124
*p*	0.124	-	0.558	0.199	0.186	0.715
**Operational**						
Correlation coefficient	0.478	−0.199	1	0.569	−0.253	−0.175
*p*	0.137	0.558	-	0.067	0.452	0.607
**Contextual**						
Correlation coefficient	0.536	0.419	0.569	1	0.092	−0.046
*p*	0.089	0.199	0.067	-	0.788	0.893
**Therapeutic**						
Correlation coefficient	0.575	0.431	−0.253	0.092	1	0.488
*p*	0.064	0.186	0.452	0.788	-	0.127
**Quality**						
Correlation coefficient	0.221	−0.124	−0.175	−0.046	0.488	1
*p*	0.514	0.715	0.607	0.893	0.127	-

## Discussion

The tool was positively approved by the CSNs who participated in the pilot study. Even though some experts regarded the tool to be quite long, this aspect was not mentioned by the CSNs. Instead, it allows the evaluation of a comprehensive amount of aspects regarding the provision of quality nursing care that is expected from the CSNs during their placement in different disciplines. Furthermore, the tool provides the opportunity for remedial actions for those CSNs who were deemed not competent by their mentors during their first meeting.

From the results of this study, it can be observed that the CSNs rated themselves very high scores in Ethics and Professional practice (4.13), Therapeutic environment (4.13) and Quality nursing care (4.16). As this might represent the Hawthorne-effect when quantitative data is measured a follow-up intervention as prescribed by the tool, such as case studies on ethics will produce more reliable results.

From the reliability test, three other competencies scored below 4.0 on the competencies overall ratings. These results confirmed that the CSNs were either novice or advanced beginners for legal practice, operational management and leadership and contextual clinical and technical competence (particularly maternity and neonatal care and mental health nursing). It should be borne in mind that a rating of 4.0 reflected a positive and significant correlation of the cited variables. These results could lead to the understanding that experience is needed in the development of a competent practitioner.

### Limitations

Limitations of the study are those characteristics of design or methodology that affected or influenced the interpretation of the results including constraints on generalisability, applications to practice, and/or utility of results (Bloemberg & Volpe 2019). The length of the developed tool and time constraints were observed to be the limitations. Some of the experts observed the developed tool to be quite long during the validation process. Hence, this is regarded as a limitation. Therefore, further research can be conducted where the focus can be on specific disciplines of the nursing profession including midwifery and mental health nursing. The developed tool was piloted to measure its reliability. This means that the tool was not exposed to the full complement of the participants, but a site of data-collection. Therefore, this is a limitation and an opportunity for further research.

## Conclusion

The aim of this article was to report the validation of CCET for CSNs in NWP, SA. In the validation phase, data presentation, analysis and interpretation was performed. These results include that of the CVI, CVR and the Cronbach’s alpha, which represent the validity and reliability measures. From the above-mentioned statements, a CCET for CSNs in the NWP, SA was refined.

## References

[CIT0001] Aiken, L.H., Sloane, D.M., Bruyneel, L., Van den Heedee, K., Griffiths, P., Busse, R. et al., 2014, ‘Nurse staffing and education and hospital mortality in nine European countries: A retrospective observational study’, *The Lancet* 383(9931), 1824–1830. 10.1016/S0140--6736(13)62631-8 PMC4035380 24581683

[CIT0002] Ayre, C. & Scally, A.J., 2014, ‘Critical values for Lawshe’s content validity ratio, measurement and evaluation in counselling and development’, *Taylor and Francis Online* 47(1), 79–86. 10.1177/0748175613513808

[CIT0003] Benner, P., 1984, *From novice to expert excellence and power in clinical nursing practice*, Addison-Wesley Publishing Company Inc., Boston, MA.

[CIT0004] Bloomberg, L.D. & Marie Volpe, M., 2019, *Completing your qualitative dissertation: A road map from beginning to end*’, 4th edn., Sage Publications, Los Angeles, CA, United State of America, viewed 16 May 2020, from https://books.google.co.za/booksaccessed2020/05/16

[CIT0005] Bracken, I., 2014, *Urban planning methods: Research and policy analysis*, Routledge Library Editions, London.

[CIT0006] Cowan, D.T., Wilson-Barnett, D.T., Norma, I.J. & Murrells, T., 2008, ‘Measuring nursing competence: Development of a self-assessment tool for general nurses across Europe’, *International Journal of Nursing Studies* 45(6), 902–913. 10.1016/j.ijnurstu.2007.03.004 17451716

[CIT0007] Creswell, J.W., 2014, *Research design: Qualitative, quantitative and mixed methods approaches*, 4th edn., Sage, Los Angeles, CA, United State of America

[CIT0008] Franklin, N. & Melville, P., 2015, ‘Competency assessment tools: An exploration of the pedagogical issues facing competency assessment for nurses in the clinical environment’, *Collegian* 22(1), 21–35. 10.1016/j.colegn.2013.10.005 26285406

[CIT0009] Gilbert, G.E. & Prion, S., 2016, ‘Making sense of methods and measurement: Lawshe’s content validity index’, *Clinical Simulation in Nursing* 12(12), 530–531. 10.1016/j.ecns.2016.08.002

[CIT0010] Gray, J.R., Grove, S.K. & Sutherland, S., 2016, *Burns and grove’s: The practice of nursing research – E-Book: Appraisal, synthesis and generation of evidence*, 8th edn., Elsevier, St. Louise, MO, viewed 16 May 2020, from https://books.google.co.za/books.

[CIT0011] Grove, S.K., Gray, J.R. & Burns, N., 2015, *Understanding nursing research – E-Book: Building an evidence-based practice*, 6th edn., Elsevier Saunders, St. Louise, MO, viewed 16 May 2020, from https://books.google.co.za/books.

[CIT0012] Hussein, R., Everett, B., Ramjan, L.M. Hu, W. & Salamonson, Y., 2017, ‘New graduate nurses’ experiences in a clinical specialty: A follow up study of newcomer perceptions of transitional support’, *BMC Nursing* 16, 42. 10.1186/s12912-017-0236-0 28775671 PMC5534089

[CIT0013] Lawshe, C.H., 1975, ‘A quantitative approach to content validity’, *Personnel Psychology*, 28, 563–575.

[CIT0014] Liou, S.R. & Cheng, C.Y., 2014, ‘Developing and validating the clinical competence questionnaire: A self-assessment instrument for upcoming baccalaureate nursing graduates’, *Journal of Nursing Education and Practice* 4(2), 56–66. 10.5430/jnep.v4n2p56

[CIT0015] Liu, M., Kunaiktikul, W., Senaratana, W.O., Tonmukayakul, O. & Eriksen, L., 2007, ‘Development of competency inventory for registered nurses in the People’s Republic of China: Scale development’, *International Journal of Nursing Studies* 44(5), 805–813. 10.1016/j.ijnurstu.2006.01.010 16519890

[CIT0016] Matlhaba, K.L., Pienaar, A.J. & Sehularo, L.A., 2019, ‘Community service nurses’ experiences regarding their clinical competence’, *Health SA Gesondheid* 24, a1284. 10.4102/hsag.v24i0.1284 PMC6917431 31934440

[CIT0017] Mohamed, L.K. & El-Sayed, K.A., 2013, ‘Junior undergraduates nurse students’ images of nursing as a career choice’, *Journal of American Science* 9(12), 25–34.

[CIT0018] Nilsson, J., Johansson, E., Egmar, A.C., Florin, J., Leksell, J., Lepp, M. et al., 2014, ‘Development and validation of a new tool measuring nurses’ self-reported professional competence – The nurse professional competence (NPC) scale’, *Nurse Education Today* 34(4), 574–580. 10.1016/j.nedt.2013.07.016 23938092

[CIT0019] Ogunyewo, O.A., Afemikhe, J.A., Ajio, D.K. & Olanlesi-Aliu, A., 2015, ‘Adolescents’ perceptions of career choice of nursing among selected secondary schools in Jos, Nigeria’, *International Journal of Nursing and Midwifery* 7(2), 21–29. 10.5897/IJNM2014.0139

[CIT0020] Persky, A.M. & Robinson, J.D., 2017, ‘Moving from novice to expertise and its implications for instruction’, *American Journal of Pharmaceutical Education* 81(9), 6065. 10.5688/ajpe6065 PMC5738945 29302087

[CIT0021] Polit, D.F. & Beck, C.T., 2017, *Nursing research: Generating and assessing evidence for nursing practice*, 10th edn., Lippincott Williams & Wilkins, London.

[CIT0022] Rajasree, J., 2016, ‘Perception towards nursing profession among higher secondary students’, *Global Journal for Research Analysis (GJRA)* 5(3), 52–53.

[CIT0023] Roets, L., Botma, Y. & Grobler, C., 2016, ‘Scholarship in nursing: Degree prepared nurses versus diploma-prepared nurses’, *Health SA Gesondheid* 21, 422–430. 10.4102/hsag.v21i0.1001

[CIT0024] Safadi, R., Jaradeh, M., Bandak, A. & Froelicher, E., 2010, ‘Competence assessment of nursing graduates of Jordanian universities’, *Nursing and Health Sciences* 12(2), 147–154. 10.1111/j.1442-2018.2009.00507.x 20602685

[CIT0025] Smit, S.C., 2018, *Ensuring research integrity and the ethical management of data*, IGI Global, Hershey, PA.

[CIT0026] South African Nursing Council (SANC), 2005, *Nursing education and training standards,* SANC, Pretoria.

[CIT0027] Wu, X., Enskär, K., Lee, C. & Wang, W., 2015, ‘A systematic review of clinical assessment for undergraduate nursing students’, *Nurse Education Today* 35(2), 347–359. 10.1016/j.nedt.2014.11.016 25497138

[CIT0028] Zamanzadeh, V., Valizadeh, L., Negarandeh, R., Monadi, M. & Azadi, A., 2013, ‘Factors influencing men entering the nursing profession, and understanding the challenges faced by them: Iranian and developed countries’ perspectives: A review article’, *Nursing and Midwifery Studies* 2(4), 49–56. 10.5812/nms.12583 25414879 PMC4228905

